# Prognostic role of vascular endothelial growth factor in hepatocellular carcinoma: systematic review and meta-analysis

**DOI:** 10.1038/sj.bjc.6605017

**Published:** 2009-04-28

**Authors:** S J Schoenleber, D M Kurtz, J A Talwalkar, L R Roberts, G J Gores

**Affiliations:** 1 Mayo Medical School, Mayo Clinic College of Medicine, Rochester, MN, USA; 2 Division of Gastroenterology and Hepatology, Mayo Clinic College of Medicine, Rochester, MN, USA; 3 Advanced Liver Disease Study Group, Miles and Shirley Fiterman Center for Digestive Diseases, Mayo Clinic College of Medicine, Rochester, MN, USA

**Keywords:** vascular endothelial growth factor, hepatocellular carcinoma, prognosis, survival

## Abstract

Hepatocellular carcinoma (HCC) is a highly vascular tumour that expresses vascular endothelial growth factor (VEGF). Various studies have evaluated the prognostic value of VEGF levels in HCC. Its overall test performance remains unclear, however. The aim was to perform a systematic review and meta-analysis of prognostic cohort studies evaluating the use of VEGF as a predictor of survival in patients with treated HCC. Eligible studies were identified through multiple search strategies. Studies were assessed for quality using the Newcastle–Ottawa Tool. Data were collected comparing disease-free and overall survival in patients with high VEGF levels as compared to those with low levels. Studies were pooled and summary hazard ratios were calculated. A total of 16 studies were included for meta-analysis (8 for tissue and 8 for serum). Methodological analysis indicated a trend for higher study quality with serum studies as compared to tissue-based investigations. Four distinct groups were pooled for analysis: tissue overall survival (*n*=251), tissue disease-free survival (*n*=413), serum overall survival (*n*=579), and serum disease-free survival (*n*=439). High tissue VEGF levels predicted poor overall (HR=2.15, 95% CI: 1.26–3.68) and disease-free (HR=1.69, 95% CI: 1.23–2.33) survival. Similarly, high serum VEGF levels predicted poor overall (HR=2.35, 95% CI: 1.80–3.07) and disease-free (HR=2.36, 95% CI 1.76–3.16) survival. A high degree of inter-study consistency was present in three of four groups analysed. Tissue and serum VEGF levels appear to have significant predictive ability for estimating overall survival in HCC and may be useful for defining prognosis in HCC.

Hepatocellular carcinoma (HCC) is the fifth most common type of cancer and the third most common cause of annual cancer-related deaths worldwide (Parkin, 2001). The incidence of HCC is predicted to increase over the next several decades as survival in patients with predisposing diseases, such as cirrhosis, is expected to increase over time (El-Serag, 2002; Marrero, 2006). Because of this, there has been great interest in evaluating factors that influence prognosis in HCC. The most widely studied prognostic factors are related to pathological characteristics of the neoplasm, including tumour size, grade, stage, and vascular invasion (Tsai *et al*, 2000; Mann *et al*, 2007; Zhou *et al*, 2007; Lam *et al*, 2008). A variety of other potential serum prognostic markers, however, remain to be further characterised (Mann *et al*, 2007).

Angiogenesis, defined as the formation of new blood vessels from existing vasculature, is an important process regulating the growth and development of malignancies including HCC (Sun and Tang, 2004; Pang and Poon, 2006). The extensive hypervascularity associated with HCC is thought to be driven in part by the pro-angiogenic factor known as vascular endothelial growth factor (VEGF) (Sun and Tang, 2004; Pang and Poon, 2006). Furthermore, the invasiveness of certain HCC lesions has recently been linked to high levels of VEGF, leading several authors to conclude that an important relationship between VEGF and prognosis exists for HCC (Li *et al*, 1998; Kanda *et al*, 2008). Conflicting data, however, have emerged regarding the ability of VEGF to predict disease progression and overall survival (OS) in HCC. This may be related to differences in the methods of measuring and reporting quantitative VEGF measurements. The three most commonly used methods are serum-based VEGF quantitation using enzyme-linked immunosorbent assay, tissue-based semi-quantitative VEGF immunohistochemistry, and tissue-based mRNA measurement (Sun and Tang, 2004). One argument against the use of serum VEGF is the confounding role of platelets, which have been reported to release VEGF into the blood (Banks *et al*, 1998; Webb *et al*, 1998). A recent study, however, found that serum VEGF alone correlated with hepatic tissue VEGF, suggesting that serum VEGF is a useful indirect marker of tumour levels (Poon *et al*, 2003).

In this study, we sought to conduct a systematic review and meta-analysis to estimate the prognostic importance of elevated serum- and tissue-based VEGF levels for OS and disease-free survival (DFS) among patients with HCC. A secondary goal of our study was to consider the methodological quality of studies examining tissue- and serum-based VEGF measurements.

## Materials and methods

### Literature search

A computer-aided literature search of PubMed (MEDLINE) 1950–present, MEDLINE in-process and non-indexed citations, EMBASE, the Cochrane Library, DARE, and ACP Journal Club was conducted. An initial search strategy using recognised search terms ((VEGF or neovascularization) and ‘prognosis’ and ‘hepatocellular carcinoma’) was conducted in July 2007 and then repeated in November 2007. Reference lists from identified primary studies were then searched to identify any studies missed by the electronic search strategies. Consultation with experts in the field was performed to further identify additional published and unpublished studies.

### Study selection

Abstracts of all candidate articles were read by two independent reviewers (DMK and SJS). Articles that could not be categorised based on title and abstract alone were retrieved for full-text review. These articles were independently read and checked for inclusion criteria. Disagreements were resolved through consensus with a third reviewer (JAT). Primary studies that reported data required for meta-analysis were identified and included. Authors of included studies were not contacted for additional, unreported data.

### Study inclusion/exclusion criteria

Inclusion criteria for primary studies were as follows: (1) proven diagnosis of HCC in humans, (2) VEGF evaluation using either serum- or tissue-based methods, and (3) correlation of VEGF with OS or DFS. There was no pre-specified sample size or follow-up period used to determine study inclusion. Language was not restricted for abstract review, but was restricted to Western languages for data collection. Studies not directly reporting hazard ratios (HRs) were allowed if data were available for statistical estimation as described below. All studies were carefully evaluated to identify duplicate patient populations. Criteria used to determine duplicate populations included study period, hospital, treatment information, and any additional inclusion criteria.

### Quality assessment of primary studies

Quality assessment was performed in each of the acceptable studies in duplicate by independent reviewers (DMK and SJS) using the Newcastle–Ottawa Quality Assessment Scale for cohort studies (Table 1) (Wells *et al*, 2003). This scale is an eight-item instrument that allows for assessment of patient population and selection, study comparability, follow-up, and outcome of interest. Interpretation of the scale is performed by awarding points, or ‘stars’, for high-quality elements. Stars are then added up and used to compare study quality in a quantitative manner. Any discrepancies were resolved by a consensus reviewer (JAT).

### Data extraction

Two reviewers (DMK and SJS) independently extracted the required information from all primary studies. Pre-specified data elements included, but were not limited to, the following: (1) demographic data regarding inclusion criteria, patient age, sex, and treatment during follow-up; (2) tumour data including number of primary lesions, size, stage, grade, vascular invasion, and metastases; (3) survival data including OS and DFS; (4) method of tissue VEGF measurement and semi-quantitative VEGF levels; and (5) method of serum VEGF measurement and average levels. Other variables included number of patients lost and reasons for patients lost during follow-up. Our primary interest was to gather data on OS, based on previously published recommendations for clinical trial end points dealing with HCC (Llovet *et al*, 2008). We did, however, also perform analyses on DFS as this was commonly reported in the studies.

### Data analysis/synthesis

After preliminary review of articles for study inclusion, inter-reviewer agreement was assessed with the kappa statistic (Cohen, 1960). Included studies were then divided into two groups for analysis: those with data regarding OS and those regarding DFS. The primary outcome for analysis was survival in patients with high VEGF values as compared to those with low VEGF values. For serum-based studies, this took into consideration a reported cut-off as determined by the authors of each study. Tissue studies usually reported data in a binary fashion, interpreting the VEGF value as either ‘high’ or ‘low.’ HRs with 95% confidence interval values were reported for individual studies, with an HR of greater than 1 being associated with an adverse outcome. Where HRs were not reported, a determination was made as to whether or not HR estimation would be possible. To be eligible for HR estimation, studies had to include the number of patients with high and low VEGF levels, along with the number of observed deaths/cancer recurrences. For studies that reported these data, mathematical HR approximation was performed using established methods (Parmar *et al*, 1998). In the case that sufficient data were not directly available but a Kaplan–Meier curve was provided, data were extracted from the survival curve and estimation of the HR was then performed using the same method.

The heterogeneity of combined HRs was initially evaluated by graphical examination of the Forrest plots. Statistical assessment was then performed using a *χ*^2^-based test of homogeneity and evaluation of the inconsistency index (*I*^2^) statistic. The *I*^2^ statistic is defined as the percentage of variability due to heterogeneity rather than chance with values >50% representing the possibility for substantial heterogeneity (Higgins *et al*, 2003). Pooled summary statistics for HRs of the individual studies were reported. Owing to *a priori* assumptions about the likelihood for heterogeneity between primary studies, the random effects model of DerSimonian and Laird (1986) was used for pooled analyses. A *P*-value of less than 0.05 was chosen for significance.

Assessment of publication bias was performed for each of the pooled study groups using the Egger's bias indicator test (Egger *et al*, 1997) defined by the following equation: SND=*a*+*b* × *s.e.(d*)^–1^. In this regression equation, SND is the standard normal deviate defined by *d* (which represents HR) divided by its standard error *s.e.(d*), *a* is the intercept, and *b* the slope. The intercept value (*a*) provides an estimate of asymmetry of the funnel plot, with positive values (*a*>0) indicating a trend towards higher HR values (ie decreased survival in patients with high VEGF levels) in studies with smaller sample sizes. All analyses were carried out using the statistical software Meta-DiSc (version 1.1.1).

## Results

**Figure 1:**
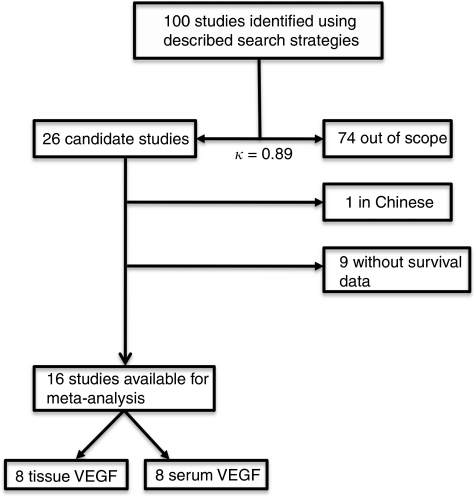
Flow chart of the meta-analysis.

The abstracts and titles of 100 primary studies were identified for initial review using search strategies as described. Reviewers identified 26 potential studies for full-text review, with excellent agreement between reviewers (*κ*=0.89). Upon further review, one article was eliminated on the basis of language of publication and another nine articles were eliminated due to inadequate data for meta-analysis (Figure 1). Given that several authors have multiple publications in this field, we took great care to ensure that data reported in each paper were unique.

Quality assessment using the Newcastle–Ottawa Scale was performed on all 16 studies included for meta-analysis. There was a tendency towards higher methodological quality in the serum VEGF studies as compared to the tissue studies (serum mean score 5.5±1.2; tissue mean score 4.6±1.4), though this did not reach statistical significance (*P*=0.20). Of note, no study attempted to control for other important prognostic factors that may have confounded the association of high VEGF with survival. Despite studies being performed at tertiary referral centres, nearly all studies (15 of 16) were performed in Asia, with one study being from Europe. Of the 16 included studies, 9 directly reported HRs; however, back-calculation from available data using statistical methods described above was necessary in 4 of 16 studies. Estimation using survival curves was required in 3 of 16 studies. During this process, data were segregated according to either OS or DFS.

The characteristics of retained studies are listed in Table 2. Studies are listed twice if they provided survival data for both DFS and OS. In total, there were 554 patients in tissue studies and 679 patients in serum studies. The median sample size for all studies was 71 patients (range=36–120). The median sample size for tissue studies was 60 patients whereas that for serum studies was 89 patients. The average age of all patients in 14 studies was 58 years (range=50–66 years). This was similar in tissue (average 56 years) and serum (average 59 years) studies. The total proportion of male subjects was around 75% in both tissue and serum studies. Data on other important prognostic markers for HCC were available in most studies. Viral hepatitis was usually reported and was available for 92% of patients in tissue studies and 79% in serum studies. Of the 16 studies, 13 reported the presence or absence of cirrhosis, with approximately 75% of patients in all studies having Child–Pugh class A or compensated disease.

Tumour size was described in most articles, but cut-offs for large tumours varied as listed in Table 2. The percentage of patients with large tumours was 48% in tissue studies and 33% in serum studies. Multiple primary lesions were observed in 15% of tissue and 23% of serum studies. Poor histological differentiation was reported in 35% of tissue studies and 38% of serum studies. Percentage of patients with vascular invasion was reported in half of the studies, but tended to be higher in serum studies (59%; range=42–80%) as compared to tissue studies (44%; range=17–82%). Overall tumour stage was reported in only six studies, but in nearly all cases VEGF levels were not determined based on stage. Although most studies required a ‘curative’ resection or treatment protocol for inclusion criteria, a number of patients inevitably had evidence of micrometastasis or regional metastatic disease on further evaluation. This was similar between studies, with 18% patients in tissue VEGF studies having evidence of local, regional, or diffuse metastatic disease as compared to 16% patients in serum studies.

Hazard ratios were recorded for each of the included studies using available data or the techniques described above. Individual studies correlated a ‘high’ VEGF level with survival data. This VEGF cut-off was chosen using different methods in each study. Although some studies, such as many of the tissue studies, used a purely binary system (positive or negative) for final analysis, other studies used a quantitative system. Many of the quantitatively based studies used the median level as a cut-off value. Still others tried to optimise the value of VEGF through examination of an ROC curve. The method used to determine the VEGF cut-off and the number of patients above this threshold is further described in Table 2.

### Summary estimates of primary studies

Studies were divided into tissue and serum groups, with survival analysis evaluating DFS and OS. In total, there were four tissue OS studies (Chow *et al*, 1997; Jeng *et al*, 2004a; Deli *et al*, 2005; Sheen *et al*, 2005), six tissue DFS studies (Jeng *et al*, 2004a; Sheen *et al*, 2005; Guo *et al*, 2006; Wada *et al*, 2006; Zhang *et al*, 2006; Ho *et al*, 2007), seven serum OS studies (Poon *et al*, 2001, 2004a, 2004b, 2007; Chao *et al*, 2003; Kim *et al*, 2004; Jeng *et al*, 2004b; Treiber *et al*, 2006), and five serum DFS studies (Poon *et al*, 2001, 2007; Chao *et al*, 2003; Jeng *et al*, 2004b; Treiber *et al*, 2006). Pooled HRs were then calculated for all groups.

**Figure 2:**
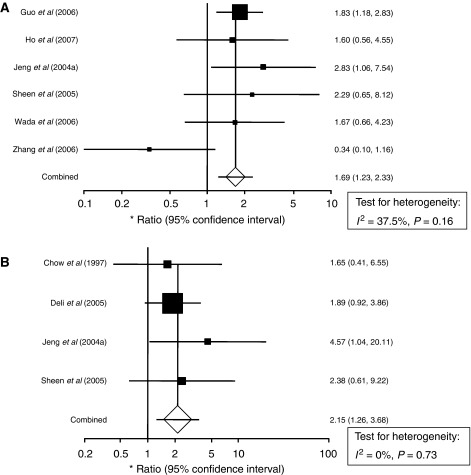
Forrest plots and meta-analysis of studies evaluating hazard ratios of high tissue VEGF levels as compared to low levels. Survival data are reported as (**A**) disease-free survival (DFS) and (**B**) overall survival (OS).

For studies evaluating tissue VEGF levels in HCC, there did not appear to be any major qualitative evidence for heterogeneity between HRs as assessed by inspection of Forrest plots for either DFS or OS (Figure 2). Increased tissue VEGF levels were significantly correlated with OS with a pooled HR estimate of 2.15 (95% CI: 1.26–3.68). There was no evidence for heterogeneity of the four available studies (*I*^2^ statistic=0%, *P*=0.73). The pooled HR estimate for DFS of the six tissue studies was 1.69 (95% CI: 1.23–2.33), with no evidence for significant heterogeneity between studies (*I*^2^ statistic=37.5%, *P*=0.16).

**Figure 3:**
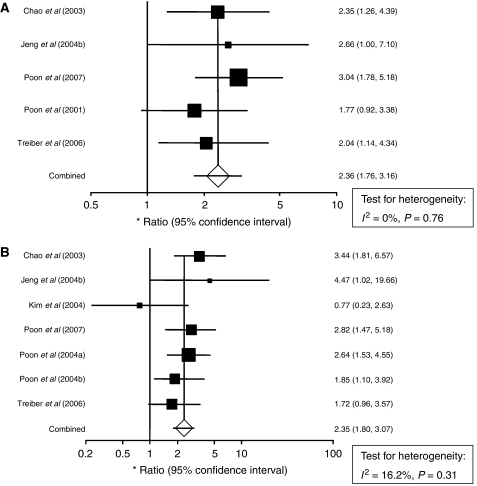
Forrest plots and meta-analysis of studies evaluating hazard ratios of high serum VEGF levels as compared to low levels. Survival data are reported as (**A**) disease-free survival (DFS) and (**B**) overall survival (OS).

Serum VEGF levels in HCC were also combined for analysis relating high levels with DFS and OS. Inspection of Forrest plots did not reveal substantial heterogeneity (Figure 3). OS in the serum studies revealed a pooled HR of 2.35 (95% CI: 1.80–3.07). Again, there was not a significant degree of heterogeneity between the seven serum studies (*I*^2^ statistic=16.2%, *P*=0.31). The pooled HR estimate for DFS was 2.36 (95% CI: 1.76–3.16). There was no heterogeneity between these five studies (*I*^2^ statistic=0%, *P*=0.76).

There was also no difference in analytic outcomes when examining serum and tissue VEGF studies based on choice of treatment (surgical *vs* non-surgical) for HCC (data not shown).

### Assessment of publication bias

**Figure 4:**
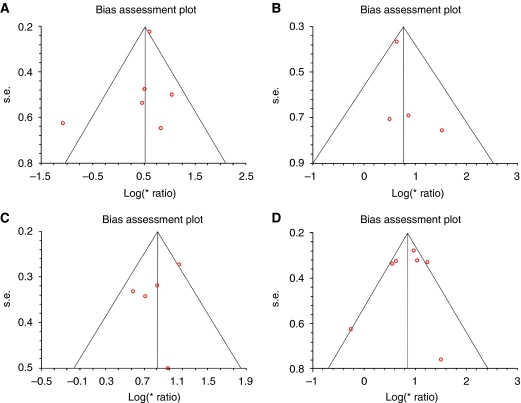
Bias assessment plots for studies included in all four meta-analyses. Plots are arranged as follows: (**A**) tissue VEGF disease-free survival, (**B**) tissue VEGF overall survival, (**C**) serum VEGF disease-free survival, and (**D**) serum VEGF overall survival.

Visual assessment of a funnel plot provided no evidence of overt publication bias for studies in each of the four pooled groups (Figure 4). Formal evaluation using Egger's test also failed to reveal evidence for significant publication bias in tissue OS (*P*=0.45) and DFS (*P*=0.56) studies. Similarly, there was no evidence for significant publication bias in serum OS (*P*=0.62) and DFS (*P*=0.69) studies.

## Discussion

There has been great interest in identifying prognostic markers for patients with HCC as these markers can help guide clinical decision-making regarding therapy and outcomes. To gain as much information as possible, it is beneficial to study a variety of prognostic measures using available demographics, pathological characteristics, and biomarker studies. In this paper, we examine the correlation of high levels of VEGF with OS after curative treatment of HCC. From our systematic review and meta-analysis, we identified and evaluated 16 primary studies from the published literature (8 for tissue and 8 for serum) comparing survival data in patients with high and low VEGF levels. Summary estimates showed that both high tissue and serum VEGF correlated to OS as well as DFS.

Quality assessment tools are being developed for prognostic studies to help identify study bias and causes for heterogeneity when performing meta-analysis. In this case, we chose to use the Newcastle–Ottawa Quality Assessment Scale, which has been validated for assessing nonrandomised case–control and cohort studies with good inter-rater reliability (Wells *et al*, 2003). In this meta-analysis, this scale allowed us to thoroughly consider the most important aspects of each study that might confound the role of high VEGF. In particular, we evaluated each study for the representativeness of the patients with HCC, recorded whether the study controlled for any key adverse prognostic factors (such as micrometastasis or age) and evaluated whether there was adequate follow-up over a long period. As this is a relatively new tool, there is not much information about what score constitutes a high-quality study *vs* a low-quality one. Therefore, we used this tool principally for comparative purposes and identified that both serum and tissue studies had features associated with the conduct of quality prognostic studies. In our meta-analysis, serum-based studies tended to be of slightly higher methodological quality than tissue-based studies although this was not statistically significant.

When comparing the results of tissue- *vs* serum-based studies, several key differences were observed. As discussed, both the study characteristics of serum studies were of higher quality as compared to tissue studies. Importantly, data from the serum VEGF studies appear to be generalisable to all patients with HCC, as the included populations were treated using a variety of curative therapies. Although tissue studies only included surgically treated patients, serum studies included patients treated with surgical or medical management, chemoembolisation, or radiofrequency ablation. When VEGF levels for both surgically and non-surgically treated groups were examined, no difference was found between groups. This suggests that choice of therapy was not potentially associated with serum VEGF levels. Furthermore, it is recognised that many factors contribute to the survival of patients with HCC, including stage, extent of cirrhosis, and treatment methods. Unfortunately, tumour stage data were not reliably reported in studies, so we could make no determination of the influence of disease stage related to VEGF level and OS. Typically, meta-regression would be carried out to control for the most important factors affecting survival. In this study, however, meta-regression techniques were not performed due to the small number of primary studies available for analysis.

It has been well documented that overall serum VEGF levels are correlated with blood platelet levels (Poon *et al*, 2001, 2003; Kim *et al*, 2004). To account for the variable influence on serum VEGF level, it has been suggested that measuring the VEGF to platelet ratio may be of value for determining the ‘true’ serum VEGF level (Poon *et al*, 2001; Kim *et al*, 2004). The studies included in this meta-analysis rarely reported data on platelet levels, and most did not make an effort to control for platelet counts when calculating survival data. However, even in the absence of this correction, serum VEGF appears to be a reasonable indirect marker of overall tissue VEGF levels (Poon *et al*, 2003). Importantly, seven of eight serum studies reported using centrifugation and sample freezing for VEGF determination. Similar collection methods have been shown to minimise the contribution of platelet VEGF to overall serum VEGF levels (Ferrero and Colombo, 2006).

Of 16 studies, 15 were performed using cohorts from medical centres in Asia. Although not entirely unexpected given the high burden of HCC in Asian populations (El-Serag, 2002), this raises a question regarding external validity of results and applicability to Western societies. However, multiple tissue (Jeng *et al*, 2004a; Sheen *et al*, 2005; Ho *et al*, 2007) and serum (Poon *et al*, 2001, 2004a, 2004b) studies failed to find a relationship between chronic viral hepatitis status and VEGF levels. This is somewhat reassuring given the high prevalence of this liver disease aetiology in Asia. Notably, when controlling for important aetiological factors of HCC, it has also been shown that overall patient survival is similar across continents (Esnaola *et al*, 2003), suggesting that our results could be applicable to various geographic populations.

Though we did not detect significant heterogeneity or publication bias between studies, it is important to note that because of the small number of primary studies analysed in each group the power to detect potentially important differences is limited. Although we did not observe significant heterogeneity between groups, heterogeneity was moderately high at 37.5% (*P*=0.16) in the tissue VEGF DFS group. This is most likely due to results from the Zhang study, which evaluated disease recurrence in patients who had undergone liver transplantation for HCC. Because the entire liver is removed during the transplantation process, the mechanisms for recurrence of the tumour may differ slightly. Potential hypotheses to explain the difference in results with this population include extrahepatic recurrence and immunosuppression. The relationship between VEGF production and these two variables is not known and requires further study.

Publication bias remains a major problem in assessing the validity of clinical research studies. Although the power to detect publication bias is reduced when using a small number of primary studies, Egger's test was chosen for our investigation because of the absence of significant heterogeneity between our primary studies. In turn, we did not find evidence that publication bias may be significantly influencing our results. We also focused on using one statistical approach, as performing multiple tests can generate discordant results that confuse data interpretation (Ioannidis and Trikalinos, 2007). Several reports have emerged proposing improvements in the reporting of prognostic studies of cancer (McShane *et al*, 2005). Criteria including (1) blinded assessment of prognostic marker to patient outcome, (2) prospective study design, (3) study time period, (4) precise outcome definition, (5) provision of candidate variable list, and (6) adequate description and references for assay methods are now being recommended. Future studies employing these quality criteria in a prospective fashion are anticipated to determine the effects of improved reporting (Kyzas *et al*, 2007).

With serum VEGF serving as an acceptable indirect marker of tissue levels, the question remains whether to consider tissue VEGF as a prognostic marker at all. At best, tissue VEGF is a qualitative test that requires interpretation by a pathologist or laboratory technician, which can be highly variable. Direct VEGF staining is only performed on a small tissue sample, introducing the possibility of sampling error given the heterogeneity in angiogenesis distribution commonly seen in tumours. Furthermore, the examination of tissue requires an invasive biopsy that can be costly. Although staining tumour tissue obtained at surgery may be of some benefit, the advantage of serum VEGF is that it can be easily drawn in a non-invasive fashion at any point in time after curative treatment. Thus, there is no need for serial biopsies and interpretation is more reliable given its quantitative nature.

On the basis of the results of this analysis, we believe serum VEGF to be a more useful test than tissue VEGF for the prognosis of HCC. Additional investigation is needed to characterise the performance of serum VEGF as a prognostic tool in patients from Europe and North America for assessing generalisability of results. Future directions could also emphasise the identification of optimal cut-points for high *vs* low VEGF levels, and determine the contribution of underlying cirrhosis to both VEGF levels and OS (Llovet *et al*, 2008). Although briefly discussed in one study (Treiber *et al*, 2006), additional investigations evaluating serial VEGF levels as a marker of disease progression or response to therapy would be a valuable addition to the literature. In the interim, this meta-analysis appears to initially support the hypothesis that high levels of VEGF are associated with a reduced probability of OS from HCC.

## Figures and Tables

**Table 1:** Newcastle–Ottawa quality assessment scale^a^

*Selection*
(1) Representativeness of the exposed cohort
(a) Truly representative of the average ‘HCC patient’ in the community (1 star)
(b) Somewhat representative of the average ‘HCC patient’ in the community (1 star)
(c) Selected group of users (e.g. nurses, volunteers)
(d) No description of the derivation of the cohort

(2) Selection of the non-exposed cohort
(a) Drawn from the same community as the exposed cohort (1 star)
(b) Drawn from a different source
(c) No description of the derivation of the non-exposed cohort

(3) Ascertainment of exposure (*Proof of HCC and VEGF measurement*)
(a) Secure record (eg surgical records) (1 star)
(b) Structured interview (1 star)
(c) Written self-report
(d) No description

(4) Demonstration that outcome of interest was not present at start of study
(a) Yes (1 star)
(b) No

*Comparability*
(1) Comparability of cohorts on the basis of the design or analysis
(a) Study controls for ‘metastasis or micrometastasis’ (1 star)
(b) Study controls for any additional factor (1 star) (*Age, stage, grade etc.*)

*Outcome*
(1) Assessment of outcome (*Death or recurrence*)
(a) Independent blind assessment (1 star)
(b) Record linkage (1 star)
(c) Self-report
(d) No description

(2) Was follow-up long enough for outcomes to occur? (*Death or recurrence*)
(a) Yes (‘2 years’) (1 star)
(b) No

(3) Adequacy of follow-up of cohorts
(a) Complete follow-up – all subjects accounted for (1 star)
(b) Subjects lost to follow-up unlikely to introduce bias – small number lost ‘(25%)’ or description provided of those lost (1 star)
(c) Follow-up rate ‘<75%’ and no description of those lost
(d) No statement

a A study can be awarded a maximum of one star for each numbered item within the Selection and Outcome categories. A maximum of two stars can be given for Comparability. Underlined and quoted phrases are provided in the scale to allow for adjustment to particular studies. Italicised phrases indicate our interpretation of the question relevant to this study.

**Table 2:** Summary table of the meta-analysis

	**Study design**	**Treat-ment**	**Number of patients (M/F)**	**Tumour grade I/II (III/IV)**	**Number with large tumours (cut-off used; cm)**	**Number with multiple primary tumours**	**Study quality points**	**VEGF detection method**	**Survival analysis**	**Hazard ratios**	**Method to determine ‘high’ VEGF cut-off level**	**Number of patients with high VEGF**	**Summary results**
*Tissue-based studies*													
Guo *et al*, 2006	P	S	90 (78/12)	65 (25)	53 (5)	15	6 of 9	Antibody	DFS	Reported in text	⩾10% staining	69	Positive
Ho *et al*, 2007	C	S	71 (52/19)	51 (19)^*^	23 (5)	6	4 of 9	Antibody	DFS	Estimated	Median	35	Indeterminate
Jeng *et al*, 2004a	C, P	S	50 (31/19)	16 (34)	38 (3)	31	6 of 9	mRNA	DFS	Estimated	NR	25	Positive
Sheen *et al*, 2005	C, P	S	60 (35/25)	14 (36)	43 (3)	31	4 of 9	mRNA	DFS	Reported in text	⩾0.500	49	Indeterminate
Wada *et al*, 2006	R	S	60 (45/15)	33 (27)	33 (3)	NR	3 of 9	Antibody	DFS	Survival curves	Strong staining	12	Indeterminate
Zhang *et al*, 2006	R	LT	82 (78/4)	NR	42 (5)	32	3 of 9	Antibody	DFS	Reported in text	>10% staining	NR	Indeterminate
Chow *et al*, 1997	R	S	36 (32/4)	24 (3)^*^	NR	NR	5 of 9	Antibody	OS	Survival curves	Any staining	13	Indeterminate
Deli *et al*, 2005	C, P	S	105 (79/26)	92 (13)	34 (5)	0	6 of 9	Antibody	OS	Survival curves	⩾60% stained	72	Indeterminate
Jeng *et al*, 2004a	C, P	S	50 (31/19)	16 (34)	38 (3)	31	6 of 9	mRNA	OS	Estimated	NR	25	Positive
Sheen *et al*, 2005	C, P	S	60 (35/25)	14 (36)	43 (3)	31	4 of 9	mRNA	OS	Reported in text	⩾0.5	49	Indeterminate
													
*Serum-based studies*													
Chao *et al*, 2003	C, P	S	98 (91/7)	85 (13)	42 (5)	46	6 of 9	ELISA	DFS	Reported in text	ROC curve	NR	Positive
Jeng *et al*, 2004b	C, P	S	50 (31/19)	16 (34)	38 (3)	31	6 of 9	mRNA	DFS	Estimated	NR	25	Indeterminate
Poon *et al*, 2007	P	RFA	120 (94/26)	14 (10)^*^	0 (5)	24	5 of 9	ELISA	DFS	Reported in text	Median	60	Positive
Poon *et al*, 2001	C, P	S	100 (76/24)	51 (49)	58 (5)	NR	4 of 9	ELISA	DFS	Estimated	Normal+2 s.d.	25	Indeterminate
Treiber *et al*, 2006	P	Medical	71 (51/20)	48 (7)^*^	NR	NR	4 of 9	ELISA	DFS	Reported in text	ROC curve	NR	Positive
Chao *et al*, 2003	C, P	S	98 (91/7)	85 (13)	42 (5)	46	6 of 9	ELISA	OS	Reported in text	ROC curve	NR	Positive
Jeng *et al*, 2004b	C, P	S	50 (31/19)	16 (34)	38 (3)	31	6 of 9	mRNA	OS	Estimated	NR	25	Positive
Kim *et al*, 2004	Unclear	Unclear	52 (39/13)	NR	20 (5)	NR	5 of 9	ELISA	OS	Reported in text	NR	29	Indeterminate
Poon *et al*, 2007	P	RFA	120 (94/26)	14 (10)^*^	0 (5)	24	5 of 9	ELISA	OS	Reported in text	Median	60	Positive
Poon *et al*, 2004a	C, P	S	108 (76/32)	56 (52)	63 (5)	28	7 of 9	ELISA	OS	Reported in text	Median	54	Positive
Poon *et al*, 2004b	C, P	TACE	80 (72/8)	NR	NR	30	7 of 9	ELISA	OS	Reported in text	Median	40	Positive
Treiber *et al*, 2006	P	Medical	71 (51/20)	48 (7)^*^	NR	NR	4 of 9	ELISA	OS	Reported in text	ROC curve	NR	Indeterminate

Summary table of studies included in meta-analysis. Study design is described as consecutive patients (C), prospective (P), or retrospective (R). Treatment describes whether the patients received curative surgical resection (S), transarterial chemoembolisation (TACE), radiofrequency ablation (RFA), or medical management of HCC. Tumour grade was most often described using the Edmondson–Steiner grading system, but occasionally other systems were utilised. For this table, studies were grouped as well/moderate (I/II) or poor (III/IV) degrees of differentiation. Incomplete data are indicated with an asterisk (^*^). Study quality is listed using the results of the Newcastle–Ottawa questionnaire (Table 1). Summary results were either positive (95% CI above 1.0) or indeterminate (95% CI crossing 1.0). NR=not reported.
